# Editorial: GPCR and G Protein-Mediated Signalling Events in the Nervous System

**DOI:** 10.3389/fncel.2022.907558

**Published:** 2022-05-03

**Authors:** Yamina A. Berchiche, Terence E. Hébert

**Affiliations:** ^1^Dr. GPCR, Malden, MA, United States; ^2^Department of Pharmacology and Therapeutics, McGill University, Montréal, QC, Canada

**Keywords:** GPCRs, cellular signaling, neuronal pharmacology, drug discovery, GPCR dimers, proteomics

In this collection in *Frontiers Research Topic*, we have put together an interesting series of articles on multiple aspects of how G protein-coupled receptors (GPCRs) and their associated G proteins signal in the nervous system. They range from describing novel drug targets, better understanding old ones to exploring how GPCRs organize into hetero-multimers as well as the analytic frameworks to capture all this meaningfully. Cellular signaling in the nervous system is complicated. While perhaps obvious, it is also true! This complexity is reflected in a need to better understand the particular contexts of cells in the brain regarding how signaling systems are arranged and how to approach them as drug targets.

One of the complexities is that GPCRs assemble into both homo- and heterodimers [reviewed in Rozenfeld and Devi (2011), Borroto-Escuela and Fuxe (2019), Sleno and Hebert (2019), Ferre et al. (2022)]. Here, we present two articles that describe the organization and function of GPCR heterodimers. In the first, Tse and Wong describe various computational approaches to capture dimerization interfaces of known heterodimers of the melatonin receptors, MT1/MT2, MT1/GPR50, MT2/GPR50, and MT2/5-HT2C using homology modeling and membrane protein docking analyses. Understanding dimer interfaces is a critical step to being able to target such heterodimers pharmacologically. Next, Lillo et al. add to our understanding of heterodimers between ghrelin and CB1 cannabinoid receptors in the striatum, demonstrating how they mutually regulate signaling driven by their respective ligands and that they are in fact upregulated developmentally in the offspring of mice exposed to a high fat diet. Much remains to be learned about the impact of GPCR homo- and heterodimerization.

Our understanding of GPCR signaling in the nervous system remains incomplete and large-scale approaches to understanding GPCR signaling are required. Degrandmaison et al. highlight the use of knock-in models to tag endogenous GPCRs for proteomic analysis. Such approaches combine all the power of modern proteomic techniques while limiting expression to endogenous levels and localizing in the correct cellular and subcellular compartments. In a distinct approach, some aspects of signaling important for neuropathic pain are described by Xie et al. It is clear that GPCRs impact Nf-kB signaling in the immune and nervous systems [reviewed in Fraser (2008) and Cattaneo et al. (2010)] and Xie et al. demonstrate that Nf-kB-mediated COX2 pathways are active in non-myelinating Schwann cells and play a role in neuropathic pain.

Next, work from Dale et al. reviews what we know about orexin signaling in the central and peripheral nervous system. They focus on understanding the complexities associated with cellular context as it relates to G protein coupling. This is a critical question the field struggles with as coupling patterns in one cell type may not always be reflected in all cell types. Similar complexities arise when considering how cells change during the progression of disease. Jones-Tabah et al. review our understanding of D1 dopamine receptor signaling and how it changes during the development of Parkinson's Disease and during therapeutic interventions to treat it. Another important neurodegenerative disease is Huntington's Disease and Komatsu reviews new approaches to treat this devastating condition, with a focus on both small interfering RNA- and antisense nucleotide-based therapies as well as targeting an orphan GPCR, GPR52, which is enriched in the striatum.

Lastly, frameworks for analyzing and interpreting GPCR signaling in distinct cellular context are critical (Kenakin, 2019; Kolb et al., in press) but what has become clear in recent years is that there is also an important temporal component to biased signaling that needs to be considered. Hoare et al. describe how events mediated by GPCRs in the nervous system can be quantified in terms of the temporal aspects of their signaling kinetics. Having a framework to do this is critical to fully exploring functional selectivity going forward.

## Author Contributions

All authors listed have made a substantial, direct, and intellectual contribution to the work and approved it for publication.

## Funding

This work was supported by grants from the Canadian Institutes of Health Research to TH (PJT-159687 and PJT-156298). TH was the holder of the Canadian Pacific Chair in Biotechnology.

## Conflict of Interest

The authors declare that the research was conducted in the absence of any commercial or financial relationships that could be construed as a potential conflict of interest.

## Publisher's Note

All claims expressed in this article are solely those of the authors and do not necessarily represent those of their affiliated organizations, or those of the publisher, the editors and the reviewers. Any product that may be evaluated in this article, or claim that may be made by its manufacturer, is not guaranteed or endorsed by the publisher.
